# Effect of Cichorium Glandulosum Extracts on CCl4-Induced Hepatic Fibrosis

**DOI:** 10.5812/ircmj.10908

**Published:** 2013-12-05

**Authors:** Dongmei Qin, Zhiping Wen, Yaru Nie, Guangmin Yao

**Affiliations:** 1 School of Pharmacy, Shihezi University, Shihezi, P. R. China; 2 Department of Pharmacy, Tongji Medical College of Huazhong University of Science & Technology, Wuhan P. R. China

**Keywords:** Chicory, Liver Cirrhosis, Chemokine CCL4, Blotting, Western, Immunohistochemistry

## Abstract

**Background:**

Cichorium glandulosum (CG), which is a Compositae family plant, is a commonly used traditional Uighur medicine capable of cleansing liver and being cholagogue, strengthening stomach, promoting digestion, inducing diuresis and reducing edema.

**Objectives:**

To study the liver and spleen indices, the levels of serum aspartate aminotransferase (AST) and alanine aminotransferase (ALT), and the histopathological changes.

**Materials and Methods:**

Rats were intragastrically administered with the extracts of a traditional Uighur medicine Cichorium glandulosum (CG). The expressions of FN, Smard3 IGFBPrPl and TGF-β1 were detected.

**Results:**

The liver and spleen indices of the CG-V group were significantly lower than those of the model group (P < 0.01). The hepatic fibrosis symptoms of the CG-V and CG-VII groups were significantly relieved, and more FN, Smard3 and IGFBPrPl were expressed than those in the normal group. The expressions of FN, Smard3 and TGF-β1 in all treatment groups were significantly higher than those in the normal group, and the expressions in the CG-V and CG-VII groups were significantly different from those in the model group (P < 0.05). Compared with the normal group, the apoptotic index of the model group was significantly higher, but the indices of the CG-V and CG-VII groups were significantly lower than that of the model group (P < 0.01).

**Conclusions:**

The extracts of CG probably exerted protective effects by influencing the TGF-β/Smads signal transduction pathway.

## 1. Background

Compositae family plant Cichorium glandulosum (CG), also known as endive, French endive and blue endive with the scientific name of Cichorium intybus L, is a biennial or perennial plant belonging to the Cichorium genus. The tender leaves, heads or roots of CG are edible or medicinal (1). Triterpenoids, lactucin and lactucopicin, as well as sesquiterpene lactones are mainly extracted from the roots of CG by using petroleum ether, ethyl acetate, and n-butyl alcohol, respectively (2).

Hepatic fibrosis is the middle pathological stage of the development from multiple chronic liver diseases, 25% to 40% of which can eventually develop into cirrhosis and even liver cancer (3). In addition, hepatic fibrosis is a repair response of liver to chronic injury and the common pathological changes of persistent hepatic injuries. Liver cells undergo sustained and repeated necrosis or inflammatory stimulation. Therefore, considerable fiber hyperplasia is accompanied by relative or absolute lack of fiber degradation, allowing the accumulation of extracellular matrix (ECM) in liver that eventually develops into hepatic cirrhosis (4, 5). Thus, blocking the onset and development of hepatic fibrosis is critical in treating chronic liver diseases.

## 2. Objectives

In this study, hepatic fibrosis rats induced by CCl4 were intragastrically administered with CG extracts prepared in our laboratory to explore the liver and spleen indices, and serum aspartate aminotransferase (AST) and alanine aminotransferase (ALT) levels, and to observe the pathological changes of liver. Immunohistochemical techniques and Western Blot were used to detect the protein expressions of FN, Smard3, IGFBPrPl and TGF-β1 to determine the anti-hepatic fibrosis effect of CG (6, 7).

## 3. Materials and Methods

The study was performed in School of Pharmacy, Shiheizi University from January 2011 to December 2012. The experiments have been approved by the Animal Ethics Committees of Shihezi University and strictly performed according to the NIH guide for the Care and Use of Laboratory Animals.

### 3.1. Experimental Animals

Male Wistar rats weighing (300 ± 20) g were purchased from Xinjiang Medical University Animal Center, License No.: SCXK (New) 2003-0001. The rats were standardly fed and allowed to drink water freely. The experiment was initiated after they adapted to breeding.

### 3.2. Drugs and Reagents

The drugs and reagents used herein include: carbon tetrachloride (CCl4, analytical grade), formaldehyde (HCHO, analytical grade), vegetable oil (commercially available soybean oil, COFCO Food Sales & Distribution Co., Ltd.), 0.9% NaCl normal saline, distilled water, petroleum ether extract (CG-V), ethyl acetate extract (CG-VI), n-butyl alcohol extract (CG-VII) (made by our Laboratory, administration dose based on human), silibinin capsule (Tianjin Tasly Pharmaceutical Co., Ltd., production batch No.: 110511), sodium pentobarbital (sold by Shanghai Chemical Reagent Procurement & Supply Station and distributed by Xinhua Chemical Plant), glutamic-oxaloacetic transaminase kit (AST, production batch No.: 20111224), glutamic-pyruvic transaminase kit (ALT, production batch No.: 20111223), Coomassie brilliant blue protein kit (production batch No.: 20111227) (all kits were purchased from Nanjing Jiancheng Bioengineering Institute), Anti-Smad3 antibody 100 μg/tube (Abcam), Anti-IGFBP7 antibody 100 μg/tube (Abcam), Anti-FN 100 μg/tube (Santa Crue Biotechnology, batch No.: G2511), Anti-TGF-β1 100 μg/tube (Abcam), 0.45 um PVDF film (Millipore, batch No.: K1HA1391LK), horseradish peroxidase-labeled goat anti-mouse IgG 0.1mL/tube (Zhongshan Golden Bridge Biotech Co., Ltd., batch No.: 101843) and horseradish peroxidase-labeled goat anti-rabbit IgG 0.1 mL/tube (Zhongshan Golden Bridge Biotech Co., Ltd., batch No.: 101305) and Tunel Roche apoptosis kit (Cat. No.: 11684817910).

### 3.3. Animal Grouping and Treatment

36 Wistar rats were randomly divided into 6 groups: a normal group, a model group, a petroleum ether extract treatment group (CG-V, 15 mg/kg), an ethyl acetate extract treatment group (CG-VI, 3 mg/kg), an n-butyl alcohol extract treatment group (CG-VII, 6 mg/kg) and a positive drug group (silibinin capsule, 8 mg/kg). Except for the normal group, the other groups were subcutaneously injected with 1.0 mL/kg 40% CCl_4_ vegetable oil solution on the back for the first time and 0.5 mL/kg each time thereafter twice a week for 12 continuous weeks. The modeling was started in the fifth week. The normal and model groups were treated with distilled water, and the other groups were intragastrically administered with 5 mL/kg CCl_4_. The experiments were continuously performed once a day for 8 weeks to establish the hepatic fibrosis model. After 12 weeks, the rats were fasted without water deprivation after the last intragastric administration, and appropriate samples were collected after 12 h.

### 3.4. Examination Items

#### 3.4.1. Organ Index Calculation

The liver and spleen were disconnected by laparotomy after the experiment, and washed with 4 °C normal saline. After absorbing excess water with filter paper, the liver and spleen were weighed to calculate the corresponding indices. Organ index = organ mass (g)/individual body mass (g) × 100 %.

#### 3.4.2. Examination of Clinical and Biochemical Indices

The blood was sampled, placed in 37 °C water bath for 1 h and centrifuged at 3.000 r/min for 15 min to separate the serum for detection. A small piece of liver tissue in the same part of the right lobe was taken, rinsed with ice brine, subjected to fat and connective tissue removing, dried with absorbent paper and weighed, and then placed in a 10 mL centrifuge tube. After adding appropriate amount of cold normal saline, the solution was homogenized at 20,000 r/min for 10 s. After 30 s of intermittence, the tissue was prepared into 10 % homogenate after being repeated three times. Then the resulting product was subjected to refrigerated centrifuge at 3,500 r/min and 4 °C for 10 min, from which the supernatant was sampled for detection. All indices were measured in accordance with the kit instructions.

#### 3.4.3. Histopathological Examination

In addition, the liver tissue in the same part of the right lobe was resected and fixed in 10% formalin solution, and then subjected to conventional HE and Masson staining to observe the histopathological changes under a light microscopy. The expressions of FN, Smard3 and TGF-β1 in liver tissues were analyzed by immunohistochemical staining (8).

#### 3.4.4. Western Blot Methods 

The expressions of FN, Smard3 and TGF-β1 in rat liver tissues were detected by the following procedure. 100 mg liver tissues were homogenized in 1 mL of lysate, and centrifuged at 4°C and 10000 r/min for 5 min. After being added in 96-well plates and stained with G250, the protein contents were measured by measuring the absorbances at 595 nm. The samples then underwent SDS-PAGE, imaging and data analysis by Gel-Pro Application.

#### 3.4.5. Cell Apoptosis Detection (TUNEL Assay)

The breakage of cell nucleus DNA in liver tissue cells at the beginning of apoptosis was detected. The color reaction of apoptosis under light microscope shows that the cells in which the nuclei were stained in dark brown were positively expressed, and those not were negative (9, 10).

Determination criteria: Five visual fields were randomly selected in each section to calculate the positive expression percentage and to collect the images under 200-fold magnification for statistical analysis. The data were expressed as (±S) and processed by SPSS 17.0. The comparisons between groups were subjected to variance analysis and t test with the inspection level of P = 0.05.

### 3.5. Statistical Analysis

The measurement data were expressed as ±S. The groups were compared by analysis of variance with the inspection level of P = 0.05. All data were analyzed by SPSS 17.0. The graphs were processed by GraphPad Prism 5.0.

## 4. Results

### 4.1. Effects of CG on Liver and Spleen Indices

Compared with the normal group, the liver and spleen indices of the model group were increased significantly. Compared with the model group, the liver and spleen indices of the CG-V, CG-VI and CG-VII groups were lowered, in which the difference between those of the CG-VI and model groups was extremely significant (P < 0.01). The liver index of the positive drug group was significantly lower than that of the model group. The results are summarized in Table 1. 

**Table 1.** Effects of CG on the Liver and Spleen Indices of Hepatic Fibrosis Rats (±S, n=6)

Group	Dose (mg/kg/day)	Liver Index	Spleen Index ^a^
**Normal**	--	0.033747 ± 3.9 E-05	0.001614 ± 9.38E-05
**Model**	--	0.036216 ± 0.000872 ^a^	0.001716 ± 6.44E-05 ^b^
**CG-V**	15	0.039165 ± 0.000417 ^d^	0.001884 ± 0.000112 ^b^^d^
**CG-VI**	3	0.036497 ± 0.003767	0.001913 ± 0.000243 ^c^
**CG-VII**	6	0.03476 ± 0.00221	0.001792 ± 7.58E-05
**Positive drug**	8	0.034223 ± 0.003596	0.001798 ± 0.000276

^a^P < 0.01, Compared with the normal group;

^b^P < 0.05;

^c^P < 0.05,

^d^P < 0.01, compared with the model group

### 4.2. Effects of CG on Serum AST and ALT Activities

Compared with the normal group, the serum AST activity of the model group was significantly elevated, indicating the modeling was successful. Compared with the model group, the levels of serum AST and ALT in the CG-V, CG-VI and CG-VII groups were decreased, in which those of the CG-VI and CG-VII groups differed significantly (P < 0.05). Besides, compared with the model group, the activities of serum AST and ALT were extremely significantly (P < 0.01) and significantly (P < 0.05) decreased in the positive drug group, respectively. The results are summarized in Table 2. 

**Table 2.** Effects of CG on the Serum AST and ALT Activities of Hepatic Fibrosis Rats ( ±S, n=6)

Group	Dose (mg/kg/d)	AST (U/L)	ALT (U/L)
**Normal**	-	82.81±14.87	123.27 ±8.04
**Model**	-	114.77±13.15 ^a^	141.25±33.15
**CG-V**	15	96.41±41.94	122.11±67.48
**CG-VI**	3	73.60±8.39 ^b^	128.38±58.14 ^b^
**CG-VII**	6	69.49±16.08 ^b^	99.75±30.56 ^b^
**Positive drug**	8	58.87±7.56 ^c^	109.05±64.33 ^b^

^a^P < 0.05, P < 0.01, compared with the normal group;

^b^P < 0.05,

^c^P < 0.01, compared with the model group

### 4.3. Histopathological Examination

#### 4.3.1. HE Staining

**Figure 1.**
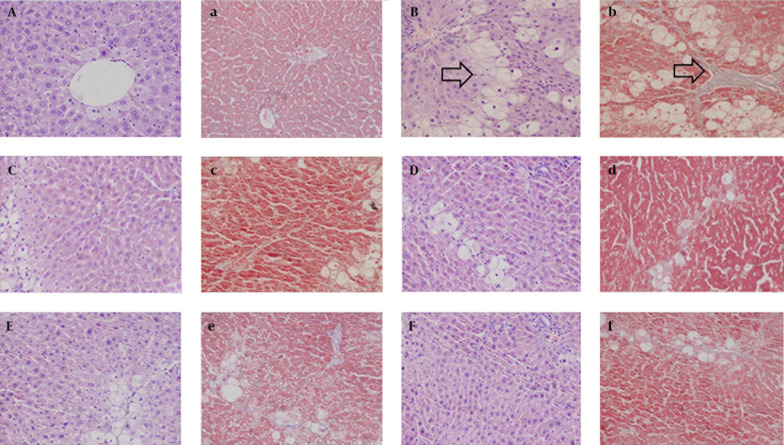
Histopathological Changes of Liver Tissues in Hepatic Fibrosis Rats (HE. Masson × 200). A) a: normal group; B) b: model group; C) c: CG-V group; D) d: CG-VI group; E) e: CG-VII group; F) f: positive drug group (A-F: HE staining; a-f: Masson staining).

In the normal group, the hepatic lobules were completely and clearly structured without abnormal hepatic sinusoids and portal areas. No lipid vacuolization was discerned, the hepatic cords were arranged orderly, no inflammatory cells formed, the cytoplasm was uniformly scattered, and the liver cells were intact without degeneration (Figure 1A). In the model group, the normal hepatic structure was damaged without obvious sinusoids, the hepatic cords were disorganized, and the cytoplasm and nucleus were incompletely structured with apparent infiltration of inflammatory cells in tissue. Ballooning degeneration and partial necrosis were also discerned in liver cells (Figure 1B). Compared with the model group, the sinusoid was more obvious, the cytoplasm of liver cells was more uniform, and the ballooning degeneration was significantly reduced in the positive drug group (Figure 1F). CG significantly mitigated the pathology of CCl _4 _-induced hepatic fibrosis in rats, alleviated the cloudy swelling of liver cells, and effectively prevented ballooning degeneration and inflammatory cells infiltration, suggesting that CG exerted protective effect on CCl _4 _-induced rat hepatic fibrosis. 

#### 4.3.2. Masson Staining

In the normal group, the liver tissue was normal without collagen fibrosis, and the liver tissue was also normally structured (Figure 1A). Compared with the liver tissue of the normal group, more collagen fibers were found in that of the model group, which led to collagen deposition and were apparently extended outward and interconnected. As a result, the normal lobular structure was jeopardized without forming false hepatic lobules (Figure 1B), indicating that the model was not evidently established. Compared with the model group, the collagen fibers in the positive drug control group were mildly extended outward from the peripheral portal area (Figure 1F). Compared with the model group, the collagen fibers in the treatment groups were significantly reduced, suggesting that CG protected the rats from CCl _4 _-induced hepatic fibrosis. 

#### 4.3.3. Immunohistochemical Staining

##### 4.3.3.1. FN

**Figure 2.**
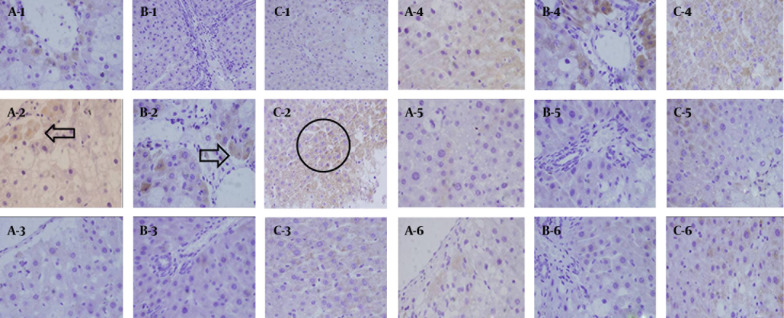
Expressions of TGF-β1, Smad3 and FN in CCl4-Induced Hepatic Fibrosis Rats. 1: normal group; 2: model group; 3: CG-V group; 4: CG-VI group; 5: CG-VII group; 6: positive drug group (a: FN, b: Smad3, c: TGF-β1).

In the normal group, FN was weakly positively expressed in the portal area, central vein area and subcutaneous sinus. The brown granules, indicate positive expression (Figure 2A), were mainly distributed in the widened portal area of the model group due to the proliferated fibrous tissues and fibrous septa. The ribbon-like shaped positively expressed brown granules isolated and surrounded the hepatic lobules (Figure 2A). The semi-quantitative results reveal that the differences between the CG-V and CG-VII groups and the model group were extremely significant (P < 0.01). The results are shown in Figure 5. 

##### 4.3.3.2. Smad3

Smad3 was weakly expressed in the normal group (Figure 2B). Smad3 protein expression was increased significantly in the model group. The brownish yellow cytoplasmic granules, indicate positive staining, were mainly located in the portal areas and fibrous septa (Figure 2B). In the treatment groups, interstitial cells, which dominated the positively expressed ones, were moderately expressed. Moreover, the treatment group was significantly less prone to positive staining than the model group. Hepatic parenchymal cells were not apparently expressed. The positively expressed cells in the portal area, portal vein and around central vein were significantly reduced compared with those in the model group. The semi-quantitative results show that the positively expressed cells in the CG-V and CG-VII groups were different from those in the model group (P < 0.01). The results are illustrated in Figure 5. 

##### 4.3.3.3. TGF-β1

**Figure 3.**
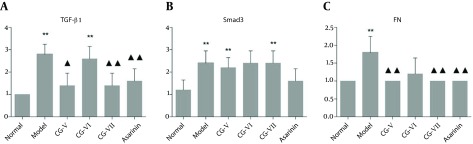
Effects of CG on the Expressions of TGF-β1, Smad3 and FN in CCl _4 _-Induced Hepatic Fibrosis Rats (n=6). **p<0.01; compared with the model group, P < 0.05, P < 0.01

Merely a small amount of TGF-β1 was positively expressed in the interstitial cells of liver tissue in the normal group (Figure 2C). In the model group, TGF-β1 expression was significantly enhanced following a brownish yellow cytosolic distribution. The staining color was significantly deepened around the lobular central vein, portal area, fibrous septa and hepatic sinusoidal Disse cavity space. Besides, they were mainly discerned in the stellate interstitial cells and inflammatory cell cytoplasm, and predominated in the portal area (Figure 2C). There was a small amount of expression in the portal area and fibrous septa of the treatment groups, and the positive staining extent was significantly reduced compared with that of the model group. Meanwhile, the positively expressed interstitial cells in the fibrous septa and the cytoplasm of inflammatory cells were decreased. The semi-quantitative results reveal that the differences between the CG-V and CG-VII groups and the model group were significant (P < 0.05, P < 0.01). The results are exhibited in Figure 3. 

### 4.4. Detection of FN, Smad3 and IGFBPrPl Expressions in liver Tissues by Western Blot

#### 4.4.1. FN

**Figure 4.**
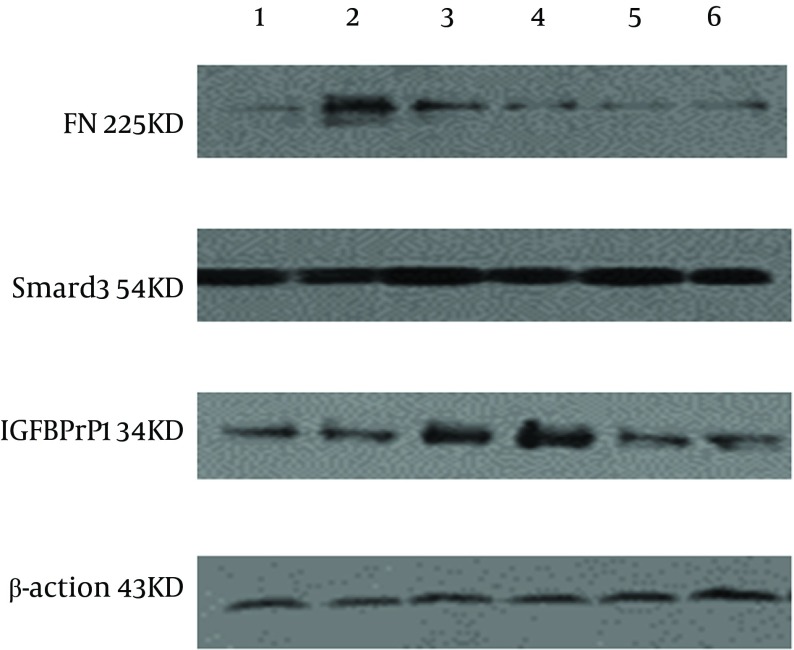
Expressions of FN, Smad3 and IGFBPrPl in CCl _4 _-Induced Hepatic Fibrosis Rats. 1: normal group; 2: model group; 3: CG-V group; 4: CG-VI group; 5: CG-VII group; 6: positive drug group.

**Figure 5.**
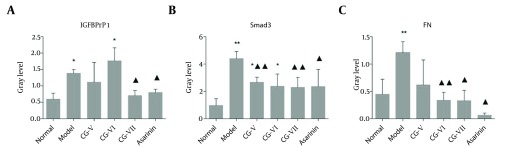
Effects of CG on the Expressions of FN, Smad3 and IGFBPrPl in CCl _4 _-Induced Hepatic Fibrosis Rats (±S, n = 6). Compared with the normal group, P < 0.05, P < 0.01; compared with the model group, P< 0.05, P < 0.01.

Significantly more FN was expressed in the model group than that in the normal group. The FN expressions in the CG-VI, CG-VII and positive drug groups were significantly lower than that in the model group (P < 0.01, P < 0.05, P < 0.05) (Figure 4 and 5). The results are displayed in Figure 5. 

#### 4.4.2. Smad3

Significantly more Smad3 was expressed in the model group than that in the normal group. The Smad3 expressions in the CG-V, CG-VII and positive drug groups were significantly lower than that in the model group (P < 0.01, P < 0.05) (Figure 4 and 5). The results are shown in Figure 5. 

#### 4.4.3. IGFBPrPl

The IGFBPrPl expression in the normal group was significantly different from that in the model group (P < 0.05), and its expressions in the CG-VII and positive drug groups were significantly lower than that in the model group (P < 0.05) (Figure 4 and 5). The results are shown in Figure 5. 

4.5. Detection of Stem Cell Apoptosis by TUNEL (TdT-Mediated dUTP Nick end Labeling)

Compared with the normal group, the apoptotic index was significantly increased in the model group, while the apoptotic indices of the CG-V and CG-VII groups were significantly lower than that of the model group (P < 0.05, P < 0.01) (Table 3). 

**Table 3.** Effects of CG on the Cell Apoptosis of CCl_4_-Induced Hepatic Fibrosis Rats (±S, n = 6)

Group	Dose (mg/kg/d)	Apoptotic Index
**Normal**	--	68.00 ± 8.36
**Model**	--	83.00 ± 8.37
**CG-V**	15	56.00 ± 20.74 ^a^
**CG-VI**	3	74.00 ± 8.94
**CG-VII**	6	66.00 ± 5.48 ^a^
**Positive drug**	8	68.00 ± 8.36 ^a^

^a^P < 0.05, P < 0.01 compared with the model group; P < 0.05, P < 0.01, compared with the normal group

## 5. Discussion

Chronic liver damage and early hepatic fibrosis animal models are classically established by CCl_4 _induction (11). The resultant models are commonly successful, highly stable, and time-saving, which can be ascribed to the pathological changes similar to the evolution of human chronic hepatitis and hepatic fibrosis. Hence, the modeling method has widely been utilized in screening anti-hepatic fibrosis drugs (12).

The experiments show that all CG extracts may reduce the activities of serum AST and ALT in hepatic fibrosis rats. The HE and Masson stained section observations exhibit normal liver tissues with distinct hepatic lobules and hepatic sinusoids. The liver tissue structure in the model group was disordered, and the liver tissue and hepatic sinusoid were replaced by a large number of connective tissues. In addition, significantly better results were detected in the treatment group than those in the model group. TGF-β/Smad3 signal pathway plays an essential role in the development of hepatic fibrosis, the blocking of which can prevent and treat hepatic fibrosis. CG extracts function in resisting to fibrosis. In this study, the expressions of four proteins closely associated with the TGF-β/Smad3 signal transduction pathway determining the pathological process of hepatic fibrosis were detected. The expressions of the four proteins in the treatment groups are decreased, which were significantly lower in the n-butanol treatment group particularly. In the meantime, n-butanol remarkably decreased the hepatocyte apoptosis index. It has previously been reported that sesquiterpene lactones were mainly extracted by n-butyl alcohol, but the pharmacologically active ingredients therein remain to be further studied. Probably, TGF-β/Smad3 signal transduction pathway may be one of the key mechanisms contributing to anti-fibrosis effect.
